# Sources of Variation Influencing Concordance between Functional MRI and Direct Cortical Stimulation in Brain Tumor Surgery

**DOI:** 10.3389/fnins.2016.00461

**Published:** 2016-10-18

**Authors:** Melanie A. Morrison, Fred Tam, Marco M. Garavaglia, Gregory M. T. Hare, Michael D. Cusimano, Tom A. Schweizer, Sunit Das, Simon J. Graham

**Affiliations:** ^1^Physical Sciences Platform, Sunnybrook Research InstituteToronto, ON, Canada; ^2^Department of Medical Biophysics, University of TorontoToronto, ON, Canada; ^3^Department of Anaesthesia, University of TorontoToronto, ON, Canada; ^4^Department of Anaesthesia, Toronto Western HospitalToronto, ON, Canada; ^5^Keenan Research Centre, St. Michael's HospitalToronto, ON, Canada; ^6^Department of Anaesthesia, St. Michael's HospitalToronto, ON, Canada; ^7^Division of Neurosurgery, St. Michael's HospitalToronto, ON, Canada; ^8^Department of Surgery, University of TorontoToronto, ON, Canada

**Keywords:** preoperative fMRI, direct cortical stimulation, behavioral testing, concordance, awake craniotomy, glioma, language, motor

## Abstract

**Object:** Preoperative functional magnetic resonance imaging (fMRI) remains a promising method to aid in the surgical management of patients diagnosed with brain tumors. For patients that are candidates for awake craniotomies, surgical decisions can potentially be improved by fMRI but this depends on the level of concordance between preoperative brain maps and the maps provided by the gold standard intraoperative method, direct cortical stimulation (DCS). There have been numerous studies of the concordance between fMRI and DCS using sensitivity and specificity measures, however the results are variable across studies and the key factors influencing variability are not well understood. Thus, the present work addresses the influence of technical factors on fMRI and DCS concordance.

**Methods:** Motor and language mapping data were collected for a group of glioma patients (*n* = 14) who underwent both preoperative fMRI and intraoperative DCS in an awake craniotomy procedure for tumor removal. Normative fMRI data were also acquired in a healthy control group (*n* = 12). The fMRI and DCS mapping data were co-registered; true positive (TP), true negative (TN), false positive (FP), and false negative (FN) occurrences were tabulated over the exposed brain surface. Sensitivity and specificity were measured for the total group, and for the motor and language sub-groups. The influence of grid placement, fMRI statistical thresholding, and task standardization were assessed. Correlations between proportions of agreement and error were also carefully scrutinized to evaluate concordance in more detail.

**Results:** Concordance was significantly better for motor vs. language mapping. There was an inverse relationship between TP and TN with increasing statistical threshold, and FP dominated the total error. Sensitivity and specificity were reduced when tasks were not standardized across fMRI and DCS.

**Conclusions:** Although the agreement between fMRI and DCS is good, variability is introduced by technical factors that can diminish the quality of patient data. Neurosurgeons should evaluate the usefulness of fMRI data while considering that (a) discordance arises primarily from FP fMRI results; (b) there is an inherent trade-off between sensitivity and specificity with fMRI statistical threshold; and (c) best results are achieved using batteries of tasks that are standardized across both mapping methods.

## Introduction

In recent years, maps of brain activity derived from functional magnetic resonance imaging (fMRI) have become more common and valued as part of the surgical management of patients diagnosed with brain tumors. Such fMRI maps may improve how the surgeon identifies high-risk eloquent areas (based on lesion-to-activation distances); assesses language lateralization; determines the optimal craniotomy extent; locates the safest surgical entry point; and selects among treatment options (e.g., craniotomy performed with or without intraoperative mapping; Lee et al., 1999; Rutten et al., 2002; Kekhia et al., 2011; Wengenroth et al., 2011; Janecek et al., 2013). Although beneficial, practical use of fMRI depends on the level of agreement (concordance) with the gold standard intraoperative brain mapping approach, direct cortical stimulation (DCS). Typically, concordance is measured according to the sensitivity (true positive rate) and specificity (true negative rate) of fMRI relative to DCS (Giussani et al., 2010; Kapsalakis et al., 2012; Meier et al., 2013). Sensitivity and specificity measures approaching 100% indicate strong agreement between fMRI and DCS. Concordance is generally good for motor mapping; high measures of sensitivity and specificity have been reported ranging from 71 to 100% and 68 to 100%, respectively (Schulder et al., 1998; Lehéricy et al., 2000; Bizzi et al., 2008; Bartoš et al., 2009; Spena et al., 2010; Meier et al., 2013). However, highly variable concordance rates have been observed across language mapping studies (Bookheimer, 2007). Amongst the broad range of language paradigms investigated in the literature, studies have reported sensitivity and specificity ranging from 59 to 100% and 0 to 97%, respectively (De Witte and Mariën, 2013).

It is very important to understand the underlying reasons for such high variability in language mapping, because such information likely can be used toward developing improved fMRI capabilities. When the existing literature is scrutinized, however, it is evident that the detailed methodology used to study concordance is not reported very often. This is unfortunate because numerous factors can potentially have important influences on the concordance rate. Examples include the rules used to classify agreement and disagreement between fMRI and DCS; the statistical threshold used to report fMRI maps of brain activity; the procedure for spatially registering fMRI and DCS maps; as well as the behavioral tasks administered (e.g., motor, language) and their underlying brain activity.

Concerning the latter factor, typical intraoperative task batteries for DCS are comprised of simple movements for motor mapping (e.g., hand clenching, foot flexing), as well as number counting and/or visual object naming tasks for language mapping (Fernández Coello et al., 2013). In comparison, the tasks performed during preoperative fMRI are more varied, without general consensus and usually without considering the tasks undertaken during DCS. The lack of task standardization between fMRI and DCS has received little attention despite the impact it may have on concordance findings (De Witte and Mariën, 2013). It is arguably even more important to consider expanding the task repertoire during DCS, given criticisms over lack of ecological validity and lack of ability to localize critical brain networks, especially during language mapping (Roux et al., 2003; Petrovich Brennan et al., 2007; Rau et al., 2007; Rofes and Miceli, 2014).

A standardized behavioral testing platform was previously developed for use across the fMRI and intraoperative environments (Morrison et al., 2015). The testing platform, equipped with a touch-sensitive tablet for writing and drawing (Tam et al., 2012), enables the use of highly similar paradigms for brain mapping during fMRI and DCS, as well as use of more flexible and sophisticated tasks in the operating room. Having used this platform in practice, here we present our concordance findings in a group of glioma patients who were subjected to standardized behavioral testing with a battery of motor and/or language tasks during preoperative fMRI and intraoperative DCS. Variability due to technical factors (i.e., use of motor vs. language tasks, matching criteria, and fMRI threshold) was explored to assess influences on fMRI and DCS concordance. Furthermore, the impact of task standardization and intraoperative task selection was investigated using patient and normative healthy control data for a traditional number counting task in contrast to more sophisticated language tasks (i.e., rhyming, phonemic word generation) delivered by the behavioral testing platform.

Toward validating use of preoperative fMRI and also refining intraoperative DCS, this work provides improved understanding of key factors that influence concordance and their relative effects, such that the sensitivity and specificity of fMRI can be better interpreted.

## Methods

### Subjects

Fourteen brain tumor patients (mean age 38.6 ± 15.7) provided written informed consent to participate in this research study with approval from the Research Ethics Boards at Sunnybrook Health Sciences Centre, Toronto, Canada, and St. MichaeŠs Hospital, Toronto, Canada. All patients had clinical or radiological evidence of a low- or high-grade glioma [World Health Organization (WHO) grade I-III] near or within eloquent brain areas associated with language and/or motor function. Patient demographics are listed in Table 1.

**Table 1:** **Patient characteristics and behavioral response(s) during intraoperative DCS mapping**.

**Patient No**.	**Age/Sex/Handedness**	**Tumor Grade/Pathology**	**Tumor location**	**Pre-operative deficit?**	**Nature and site of language/motor error**	
					**Number counting**	**Word generation (P1, P2, P4), Rhyming (P3)**
P1	38/F/R	II/Oligodendroglioma	R-frontal	No	No sites identified	Speech arrest speech apraxia
P2	23/F/R	I/Ganglioglioma	L-insular	No	Speech arrest	Speech arrest speech apraxia
P3	48/F/R	III/Gemistocytic Astrocytoma	L-fronto-insular	Anomic aphasia, sentence comprehension (reading)	Facial twitching and dysarthria (2,4)	Facial twitching, dysarthria, speech arrest and conduction aphasia (1-6)
P4	58/F/R	III/Anaplastic Oligodendroglioma	R-frontal	No	No sites identified	Speech arrest
					**Hand/Oral motor tasks**	
					Mouth twitching	
P5	25/F/L	III/Anaplastic Astrocytoma	R-frontal	No	Hand movement	
P6	35/F/R	II/Astrocytoma	L-parietal	No	Hand movement	
P7	27/M/R	II/Oligodendroglioma	R-frontal	No	Hand movement	
P8	73/M/R	III/Anaplastic Oligodendroglioma	L-frontal	No	Hand movement	

^*^Numbers in brackets for patient P3 correspond to the site(s) mapped intraoperatively in **Figure 6**.

Contraindications to MRI, and/or the presence of any other major neurological or psychological disorder were grounds for exclusion. Similar criteria (i.e., age, handedness, and sex) were used to recruit 12 patient-matched healthy controls (mean age 38.8 ± 13.0; 7 female, 5 male).

### Functional MRI

Functional MRI was performed on a research-dedicated 3T MRI system (MR750, GE Healthcare, Waukesha, WI) during a single visit to Sunnybrook Research Institute, Toronto, Canada. The protocol included IR-FSPGR (inversion recovery prepared fast spoiled gradient echo) T1-weighted axial anatomical imaging [repetition time (TR)/echo time (TE)/flip angle (θ) = 82 ms/3.2 ms/8 degrees]; field of view (FOV) = 22 × 22 cm; 190 slices; slice thickness = 1 mm, followed by multiple fMRI “runs” using a T2^*^-weighted sequence with spiral in/out k-space trajectory [TR/TE/θ = 2000 ms/30 ms/70 degrees; field of view (FOV) = 20 × 20 cm; 30 slices; slice thickness = 4.5 mm]. Patients and healthy controls performed up to 8 block-design language and motor tasks including: number counting, phonemic word generation, word copying, decision-based rhyming, semantic sentence comprehension, hand clenching, foot flexing, and tongue movement (Table 2). One task was imaged per run. Behavioral tasks were delivered and responses were recorded via an fMRI-compatible tablet system (Tam et al., 2012) composed of a touch-sensitive surface and writing stylus, controlled by E-Prime computer software (Psychology Software Tools, Sharpsburg, PA). Given the novelty of the tablet system, recruitment of healthy controls was required to generate normative datasets for each task. These data enabled comparisons with patient data, and assessment of fMRI reproducibility that is reported elsewhere (Morrison et al., 2016).

**Table 2:** **Functional MRI behavioral task battery**.

**Task**	**Contrast**	**Description**	**Initial rest period (s)**	**No. of contrast blocks**	**Total duration (s)**
Number counting	Task vs. rest	Task—covertly count from 1 to 10 at a self-controlled pace for 15 s	15	8	240
		Rest—15 s			
Phonemic word generation	Task vs. control; task vs. rest	Task—write words on tablet beginning with the presented letter for 60 s	12	3 (3 different letters)	300
		Control—write varying lengths (self-chosen) of symbol strings composed of double-loops (e.g. “8,” “88,” “888,” etc.) for 20 s			
		Rest—16 s			
Word copying	Task vs. control; task vs. rest	Task—copy the presented word(s) on tablet for 25 s	12	5	312
		Control—self-directed marking with tablet stylus (fine motor movement) for 25 s			
		Rest—10 s			
Decision-based rhyming	Task vs. control	Task—decide if the presented word pairs rhyme; respond “yes” or “no” by pressing an icon on the tablet (18 s)	12	8	300
		Control—decide if the presented line pairs are alike in volume and orientation; respond “yes” or “no” by pressing an icon on the tablet (18 s)			
Semantic sentence comprehension	Task vs. control	Task—decide if the presented sentence is semantically and grammatically correct; respond “yes” or “no” by pressing an icon on the tablet (21 s)	12	8	348
		Control—decide if the presented line pairs are alike in volume and orientation; respond “yes” or “no” by pressing an icon on the tablet (21 s)			
Hand clenching	Task vs. rest	Task—squeeze a latex squeeze toy with hand^*^ continuously at a self-directed pace for 15 s	15	8	240
		Rest—15 s			
Foot flexing	Task vs. rest	Task—flex foot^*^ up and down continuously at a self-directed pace for 15 s	15	8	240
		Rest—15 s			
Tongue movement	Task vs. rest	Task—move tongue in any arbitrary direction, continuously, at a self-directed pace for 15 s	15	8	240
		Rest—15 s			

* *The moving hand/foot (i.e., left vs. right limb) was selected according to the hemisphere of tumor dominance to maintain contralaterality*.

To generate brain activity maps, the fMRI data were preprocessed and then fitted to a General Linear Model using AFNI (Analysis of Functional Neuroimages) freeware (version:2011_12_21_1014) (Cox, 1996). Data preprocessing involved the following AFNI functions: outlier censoring and interpolation (3dDespike), physiological correction of cardiac and respiratory data (3dretroicor), motion correction (3dvolreg), slice-timing correction (3dTshift), spatial smoothing with an isotropic 6 mm Gaussian filter (3dmerge), temporal detrending (3dDeconvolve), and spatial normalization into Talairach reference space (@Auto_tlrc). Patient data were evaluated at the individual level whereas group activity maps were generated from the controls (*N* = 12). For patients, a small variable threshold, t_s_, was applied such that clusters of brain activity were most stable in volume and spatial extent. Two additional datasets, t_s_+ and t_s_- were created at ±15% of the initial threshold to assess errors associated with a fixed threshold. A thresholding method based on cluster size was applied to correct for multiple comparisons (Woo et al., 2014). Using the AFNI 3dClustSim function, contiguous active voxels forming clusters of ≥20 voxels were preserved, whereas all remaining voxels were filtered out. Group activity maps from the controls were also corrected using the same method.

To visualize fMRI results in three-dimensional (3-D) space, the T1-weighted anatomical data were segmented and surface-rendered using Freesurfer freeware (version:5.3.0) (Dale et al., 1999). Functional data were overlaid onto the 3-D surfaces using a surface mapping function (SUMA) within AFNI. Patient data were excluded in cases where brain anatomy (and consequently the surface rendering) was severely distorted by the tumor volume and/or a previous resection cavity.

### Intraoperative DCS

Awake craniotomy procedures were performed at St. Michael's Hospital, Toronto, Canada, where the patients received primary care. A unique anesthetic protocol based on a primary sedative, dexmedetomidine, in combination with a bupivacaine-based scalp nerve block, provided optimal operative conditions including the ability to conduct behavioral testing during intraoperative mapping without airway manipulation (Garavaglia et al., 2013). Asleep-awake-asleep and asleep-awake-awake anesthetic techniques were implemented according to the level of risk for postoperative deficit (based on tumor proximity to functional areas) and patient preference. The intraoperative task battery was tailored for each patient according to their tumor location, with precedence given to either motor mapping and/or language mapping as appropriate. In cases where language mapping was a priority, patients performed the traditional number counting task verbally and at least one additional language task from the preoperative fMRI task battery. If the sensorimotor cortex (i.e., pre- and post-central gyri) were surgically exposed for the language patients, then motor mapping was also performed. Task instructions were delivered using an intraoperative testing platform previously derived from the fMRI-compatible tablet system (Tam et al., 2012; Morrison et al., 2015), thus providing standardized testing conditions for data collection.

**Figure 1:**
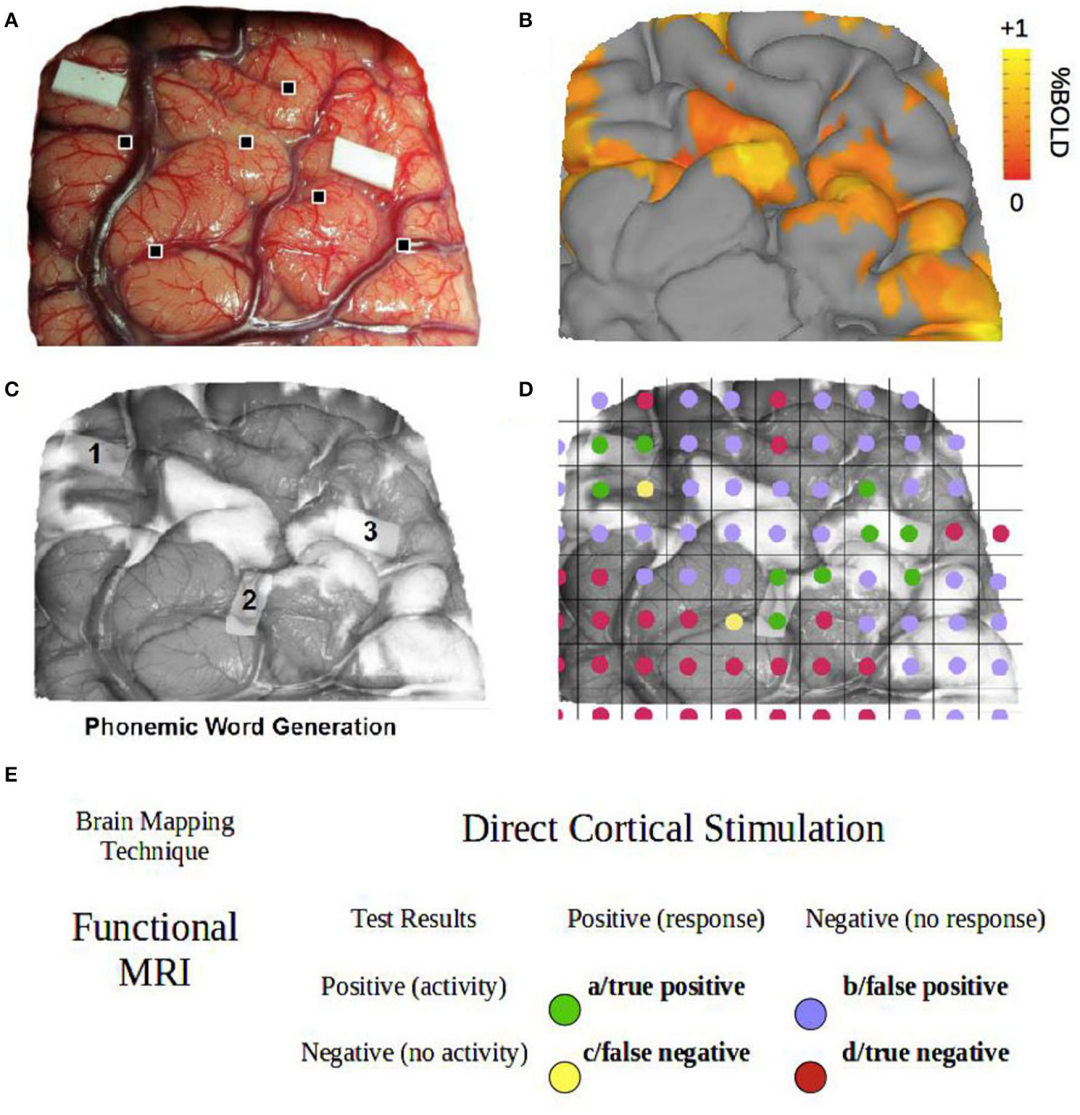
**Contingency table methods. (A)** DCS surface data for the phonemic word generation task (patient P1). Cortical landmarks are indicated by the black squares. **(B)** fMRI surface data for the phonemic word generation task (patient P1). **(C)** Co-registered fMRI and DCS surface data. **(D)** Decomposition of co-registered data into 5 × 5 mm grid squares, each color-coded according to the definitions in **(E)**. **(E)** Example of a two-by-two contingency table for fMRI vs. DCS test results.

An OCS2 Ojemann cortical stimulator (Integra LifeSciences, Plainsboro, NJ) was used at 2–6 mA to evoke inhibitory or excitatory behavioral responses during task performance. Starting at 2 mA, all gyri exposed by the craniotomy were stimulated at 5 mm spatial increments for 1–2 s, with no sites stimulated twice in succession. If there was no response to stimulation or if the response was equivocal, the stimulator intensity was incremented by 1 mA. A site was labeled “positive” with a sterile surgical chip (9 × 4 × 1.5 mm) if an inhibitory or excitatory response was repeated at least 3 times for a localized site. Cold irrigation saline was available in the event of stimulation-induced seizures. Video recordings of the intraoperative mapping procedure were simultaneously acquired from the mounted brain camera (Swann PRO-642) component of the intraoperative testing platform, as well as from a high-definition digital camera (Canon PowerShot SX50 HS) to evaluate and classify behavioral responses postoperatively. The digital camera provided high resolution images of the brain surface (with surgical chips; Figure 1A) for optimal co-registration with the anatomical MRI surface rendering (Figure 1B). In an alternative method of collecting spatial data, positives sites may be labeled on a conventional preoperative MRI volume dataset via intraoperative neuronavigational equipment. In the present study, such equipment was available (BrainLab) but was used only to identify the optimal surgical entry point and to assist with tumor resection, rather than for data collection. This was a practical decision given the error margin on manual placement of surgical chips, and also the lack of brain shift representation on preoperative anatomical MRI datasets.

### Co-registration of anatomical MRI surfaces and intraoperative photographs

The 3-D MRI surface renderings were initially rotated to locate the approximate craniotomy window, and then captured as a two-dimensional (2-D) grayscale image. Co-registration of the 2-D MRI brain surface representations and 2-D grayscale intraoperative photographs was done using the “imregister” function available within MATLAB (Statistics Toolbox, The MathWorks, Inc., Natick, MA). The “imregister” algorithm utilizes a mutual information (MI) cost function to perform the registration. This involves applying a series of affine transformations (e.g., scaling, translations, rotations, shear mapping) to the 2-D MRI brain surface representation to optimize similarity with the analogous 2-D intraoperative photograph. Each iteration of the algorithm produces an MI value, where a greater value corresponds to better co-registration.

**Figure 2:**
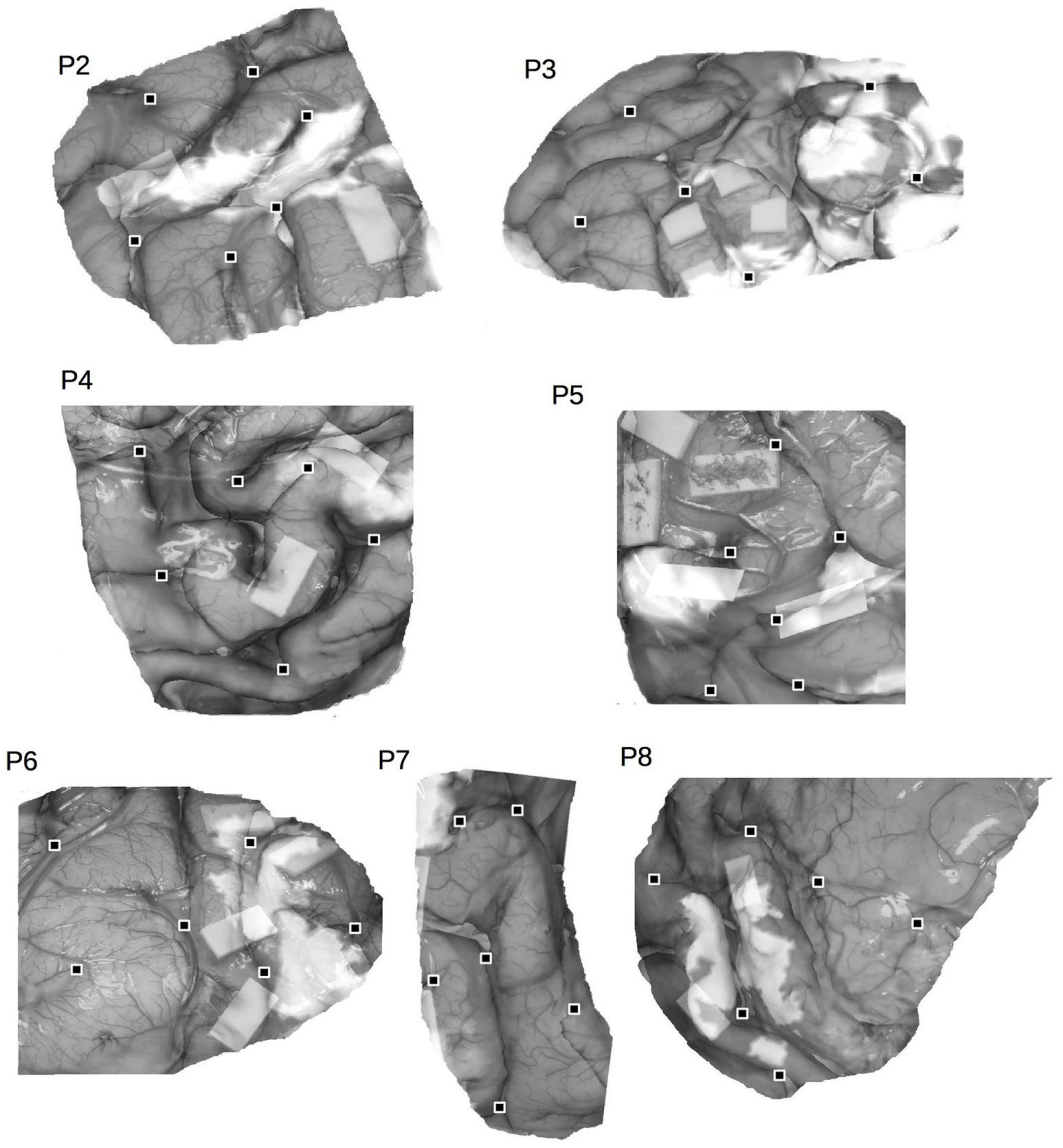
**Co-registered fMRI and DCS surface data for P2-P8**. Cortical landmarks are indicated by the black squares.

The algorithm was executed until changes in the MI value were < 0.1% across 100 iterations. For validation, six coordinate landmarks were labeled on both the MRI surface data and the intraoperative photograph. Each corresponded to a unique anatomical feature (e.g., intersecting sulci of the cerebral cortex). A Euclidian distance measure was used to quantify co-registration error and ensure that the error remained below the spatial resolution of fMRI (voxel size 3 × 3 × 4.5 mm) and DCS (approximately 5 × 5 × 5 mm). A transparency tool enabled simultaneous viewing of the fMRI and DCS results (Figures 1C, 2).

### Evaluating spatial concordance

To evaluate and measure spatial concordance between fMRI and DCS, the sensitivity and specificity of fMRI were calculated according to

(1) $$\begin{array}{l} Sensitivity=\frac{TP}{TP+FN}, \end{array}$$

(2) $$\begin{array}{l} Specificity=\frac{TN}{TN+FP}, \end{array}$$

where TP, TN, FP, and FN respectively correspond to the frequency of true positives, true negatives, false positives, and false negatives. The TP and TN frequencies represent the number of regions where fMRI and DCS both positively and negatively agreed, respectively, whereas the FP and FN frequencies respectively refer to the number of regions where fMRI was active in the absence of DCS findings, and regions where fMRI failed to produce activity when DCS was positive.

To classify the data and subsequently tabulate the frequencies, co-registered fMRI and DCS data were decomposed into a grid of 2-D pixels. Functional MRI datasets thresholded at t_s_+, t_s_, and t_s_−, were independently superimposed onto the co-registered intraoperative photograph using optimal transformation parameters such that both fMRI and DCS results for a given task were visible. Each dataset was decomposed into a grid of 5 × 5 mm pixels (Figure 1D) to mimic the intraoperative mapping trajectory given the 5 mm inter-electrode spacing of the stimulator. The results for each patient, behavioral task, and unique fMRI threshold were recorded independently in two-by-two contingency tables (Figure 1E). As the grid placement was arbitrary, five repeated measures were taken as the grid was incrementally translated in a 1 mm diagonal trajectory. Thus, for each superimposed image at t_s_+, t_s_, and t_s_−, the final contingency tables consisted of frequency values averaged across the five repeated measurements.

A series of manipulations were undertaken to explore the variation in results due to technical factors. Sensitivity and specificity were calculated directly from the contingency tables and averaged for the total group, as well as for the motor and language sub-groups to assess the effect of task. The within-patient variability associated with grid placement and statistical threshold were computed separately for motor and language. A Mann–Whitney U test at the 95% confidence interval was used to test for statistically significant differences in sensitivity, specificity, and within-patient variability values across the factors.

Receiver operating characteristic (ROC) curves (i.e., sensitivity vs. 1-specificity) were constructed from the motor and language sub-group data to assess the influence of fMRI statistical threshold on group-level concordance. To evaluate concordance in more detail, the variation was further explored by normalizing and plotting the TP, TN, FP, and FN frequency data for visualization at both the individual and group level. To normalize the data on a scale from 0 to 1, each frequency value was divided by the total number of sampled pixels (a unique number to each dataset). The TP, TN, FP, and FN contributions were ultimately represented as decimal probabilities that summed to unity. In a combined patient plot, the normalized TP frequencies were plotted against the total error (i.e., sum of FP and FN) at t_s_+, t_s_, and t_s_−. Similarly, the normalized TN frequencies were plotted against the total error. A bar plot was also generated to evaluate error contributions from FP vs. FN findings. Group level trends were delineated using regression analysis, the range(s) of variation were computed, and significance testing was performed as appropriate. This approach to further explore the data reveals the relative contributions of agreement (i.e., TP, TN) and error (i.e., FP, FN) in a manner that is easily visualized and has practical importance.

Finally, the variability due to task standardization and intraoperative task selection was explored using fMRI group activity maps in healthy controls for comparison with preoperative and intraoperative data from patients. A group activity map representing a traditional intraoperative number counting task was compared with a conjunction map of phonemic word generation and rhyming. The two latter tasks, which represent non-traditional paradigms performed by the patients, were grouped together given their similar activation patterns (Lurito et al., 2000; Morrison et al., 2016). The extent of overlap (number counting compared to word generation and/or rhyming) was quantified using the Jaccard similarity coefficient. The sensitivity and specificity were measured based on representative patient case data for the conditions where preoperative and intraoperative testing paradigms were (1) highly similar (e.g., standardized; number counting vs. number counting) and (2) distinctly different (e.g., non-standardized; phonemic word generation; and rhyming vs. number counting).

## Results

### Patient cohort

All 14 patients complied well with task instructions (Table 2), successfully undergoing both preoperative fMRI and intraoperative DCS during an awake craniotomy procedure. Six patients were initially identified for language mapping, seven for motor mapping, and one for both language and motor mapping. Three language and three motor patients were subsequently excluded due to significant distortion of the brain surface anatomy affecting data co-registration. Tumor infiltration of the pia matter was the primary source of distortion in 4 of the excluded patients, while the remaining two patients had previous resection cavities distorting the brain surface. For the remaining 8 patients, co-registration results are presented in Figure 2. Demographics are listed in Table 1, including the intraoperative tasks performed, the nature of language/motor errors induced by stimulation, and any language/motor deficit(s) identified on clinical examination. Language mapping typically induced inhibitory-like responses (e.g., speech arrest), whereas motor mapping resulted in excitatory-like behaviors (e.g., involuntary hand movement). Only one patient (P3) presented with a preoperative behavioral deficit, affecting language.

Group activation maps from the healthy control subjects revealed peak regions of activity for the rhyming and phonemic word generation task localized to the left hemisphere, including: precentral gyrus (Brodmann area BA 6), medial frontal gyrus (BA 6), superior frontal gyrus (BA 6), middle frontal gyrus (BA 9, 46), inferior frontal gyrus (BA 45), and superior temporal gyrus (BA 22). Apart from BA 45, the same regions were also activated by the number counting task, though to a lesser spatial extent. Hand and oral motor activations were localized along the precentral gyrus, as expected. Due to differences in patient tumor location and craniotomy extent, there was variation in the number of peak fMRI regions actually exposed during stimulation.

### Co-registration

Figures 1A, 2 show co-registration results for patients P1-P8. Co-registration was excellent overall, requiring approximately 500 iterations on average to reach the specified convergence criterion. The average displacement of surface anatomical landmarks was 0.92 ± 0.31 mm across subjects, well within the spatial resolution of the two brain mapping techniques.

### Sources of variation influences fMRI and DCS concordance

#### Sensitivity, specificity, and within-patient variability

Group sensitivity and specificity measures are reported in Table 3. Concordance values were relatively high and similar in magnitude over all patients; the average sensitivity was 0.75 ± 0.16, whereas the average specificity was 0.77 ± 0.14. Averaging within the task sub-groups (i.e., motor, language) revealed higher concordance values and lower between-patient variability for motor mapping (sensitivity: 0.85 ± 0.08; specificity: 0.81 ± 0.07) vs. language mapping (sensitivity: 0.66 ± 0.16; specificity: 0.74 ± 0.16). The difference in concordance for motor and language mapping was statistically significant for sensitivity (p < 0.05), but not for specificity. For language mapping, no significant differences were found between number counting (sensitivity: 0.60 ± 0.10; specificity: 0.87 ± 0.07) and word generation/rhyming (sensitivity: 0.69 ± 0.19; specificity: 0.62 ± 0.14). Nonetheless, trends showed greater sensitivity for the latter and greater specificity for the former.

**Table 3:** **Group sensitivity and specificity measures**.

**Group**	**Sensitivity ±$\bar{\sigma }$**	***P*-value (95% confidence interval)**	**Specificity ±$\bar{\sigma }$**	***P*-value (95% confidence interval)**
Total Group	0.75 ± 0.16	N/A	0.77 ± 0.14	N/A
Motor	0.85 ± 0.08	**0.03**	0.81 ± 0.07	0.72
Language	0.66 ± 0.16		0.74 ± 0.16	
Number counting	0.60 ± 0.10	0.80	0.87 ± 0.07	0.11
Word generation/Rhyming	0.69 ± 0.19		0.62 ± 0.14	

*Bold values are statistically significant*.

The impact of grid placement and fMRI threshold on concordance is reported in Table 4 using the average standard deviation, $\bar{\sigma }$. Across patients, grid placement influenced sensitivity values (${\bar{\sigma }}_{motor}=0.11;;\;{\bar{\sigma }}_{language}=0.10)$ more than specificity values$\;\left({\bar{\sigma }}_{motor}=0.05;;\;{\bar{\sigma }}_{language}=0.04\right)$, irrespective of motor or language task. For fMRI threshold, the impact on sensitivity was slightly greater for motor tasks $\left(\;\bar{\sigma }=0.1\right)$ than for language tasks $\left(\;\bar{\sigma }=0.06\right)$, whereas the opposite effect was observed for specificity ($\bar{\sigma }$ values of 0.04 and 0.09, respectively). The latter comparison was almost statistically significant (*p* = 0.06).

**Table 4:** **Average standard deviation(s) in sensitivity and specificity due to sources of variation**.

**Source of variation**		**Average $\bar{\sigma }$ in sensitivity**	***P*-value (95% confidence interval)**	**Average $\bar{\sigma }$ in specificity**	***P*-value (95% confidence interval)**
Grid placement	Motor	0.11	0.44	0.05	0.58
	Language	0.10		0.04	
fMRI threshold	Motor	0.10	0.33	0.04	**0.06**
	Language	0.06		0.09	

*Bold values are statistically significant*.

#### Correlations between agreement and error

**Figure 3:**
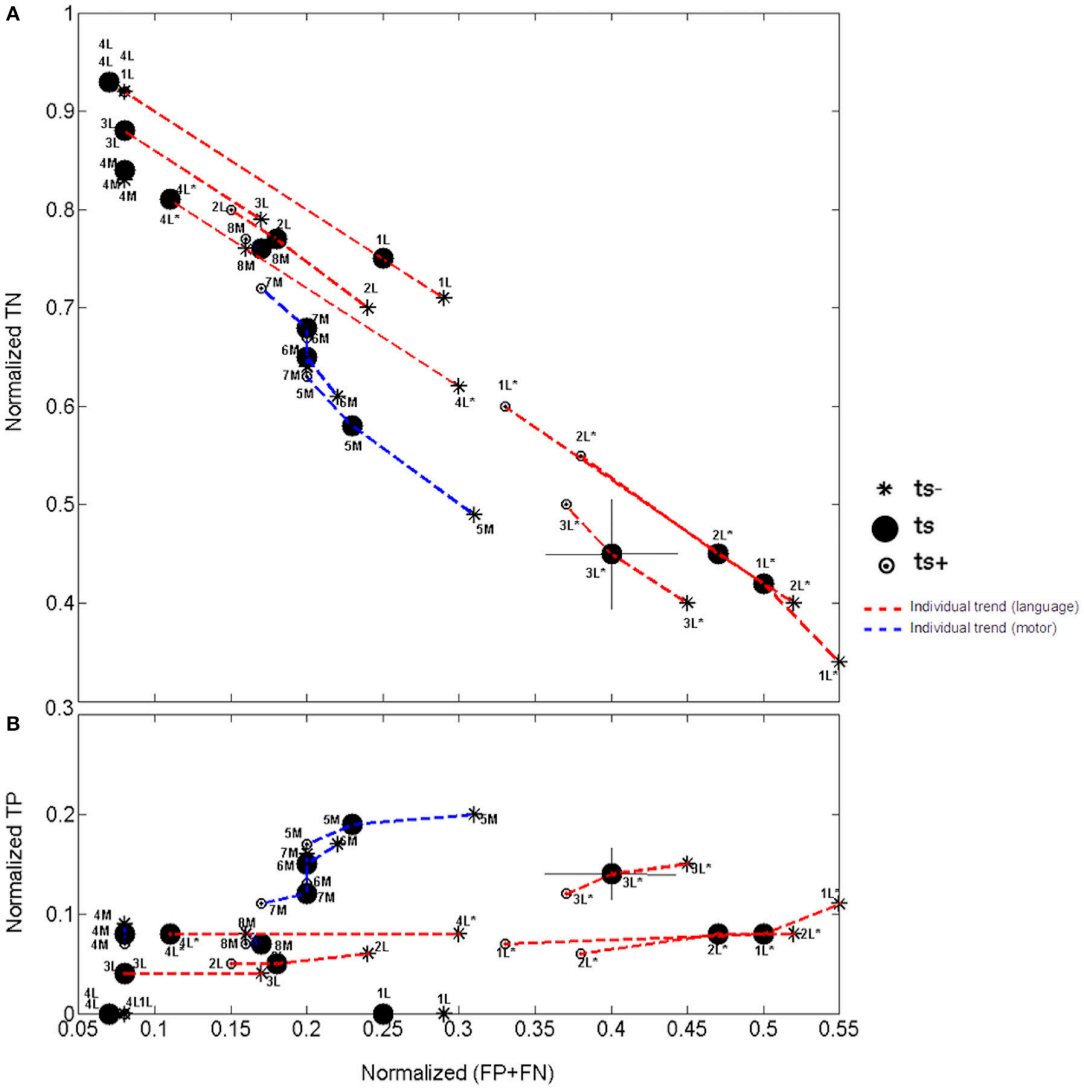
**(A)** Normalized true negative agreement vs. the total error. **(B)** Normalized true positive agreement vs. the total error. Patients are labeled according to Table 1, with motor and language tasks distinguished by letters “M” and “L,” respectively. Word generation/rhyming are distinguished from number counting by an asterisk. Group error associated with grid placement is denoted by crosshairs (see patient 3L^*^). Note the continuous scale on the y-axis.

The patient data in Figure 3 show that for motor mapping with fMRI and DCS, there is 7–20% (mean 12 ± 5%) probability of TP occurrences over the group, an even greater 49–84% (mean 70 ± 10%) probability of TN occurrences, and 8–31% (mean 18 ± 6%) chance of error (either FP or FN agreement). For language mapping, the data show a 0–15% (mean 6 ± 4%) probability of TP agreement (zero probability corresponds to cases where DCS did not produce a language response), a 34–93% (mean 68 ± 20%) probability of TN occurrences, as well as a 7–55% (mean 26 ± 16%) chance of error.

Interesting relationships between these quantities are observable at the individual patient level, as shown in Figure 3 by plotting normalized TP and TN values on a continuous scale vs. normalized total error (FP+FN) across language and motor tasks for each patient. The impact of grid placement and fMRI threshold are also shown in this figure for additional perspective. Considering first the relationship between normalized TN and normalized error (Figure 3A) with fixed fMRI threshold (t_s_) and grid placement, a negative correlation is evident overall: as the TN values decrease across patients, total errors also increase. In contrast, a positive correlation is observed for normalized TP vs. normalized total error (Figure 3B): the TP value increases across patients as errors increase. As indicated by the colored lines and symbols, some differences in these correlations are observable across motor tasks (blue) and language tasks (red). The negative correlation between TN and error is very similar for both motor and language tasks (Figure 3A), whereas the positive correlation between TP and error (Figure 3B) is slightly more pronounced for motor tasks, indicating approximately twice the TP value for a given error level. Regression analysis confirmed these observations, revealing similar slope, *m*, and R-squared values in Figure 3A for motor and language (*m*_motor_ = −1.2, $R_{\text{motor}}^{2}\;=$ 0.96; *m*_language_ = −1.6, $R_{\text{language}}^{2}\;=$ 0.89), and greater values for motor than language for Figure 3B (*m*_motor_ = 0.65, $R_{\text{motor}}^{2}\;=$ = 0.57; *m*_language_ = 0.15, $R_{\text{language}}^{2}\;=$ 0.34). However, differences in the latter comparison were not statistically significant (*p* = 0.08).

**Figure 4:**
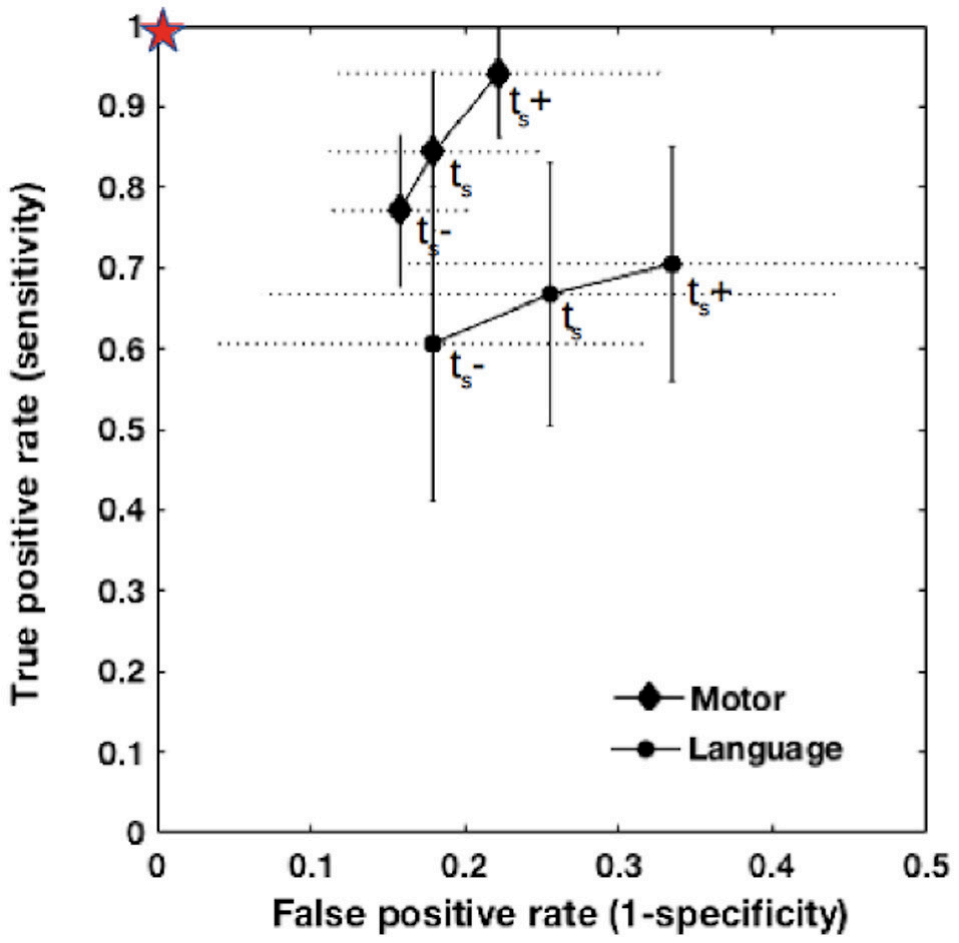
**ROC curves for the agreement between fMRI and DCS at t_**s**_−, t_**s**_, and t_**s**_+**. Vertical error bars (solid line) and horizontal error bars (dotted line) are shown for each data point representing a group mean for language or motor mapping. The red star indicates the ideal point of perfect agreement where sensitivity and specificity are maximized.

The average normalized errors associated with grid placement were 0.02, 0.06, 0.03, and 0.01 across patients for the absolute TP, TN, FP, and FN values, respectively, independent of task. For clarity, this source of error is shown for the two plots in Figure 3 by crosshairs at a single data point (t_*s*_, 3L^*^). The effect of increasing the fMRI threshold (through t_s_−, t_s_, and t_s_+) is also shown in Figure 3 using dashed lines, and furthermore in Figure 4 using a traditional ROC plot. As the threshold increased, the normalized TP value (and therefore sensitivity) decreased; the normalized TN (and therefore specificity) increased, and the total error decreased. The net effect of these changes did not have significant impact on the positive and negative correlations shown in Figures 3A,B, respectively, but predominantly caused a collective shift of the data for all patients along the lines of regression.

**Figure 5:**
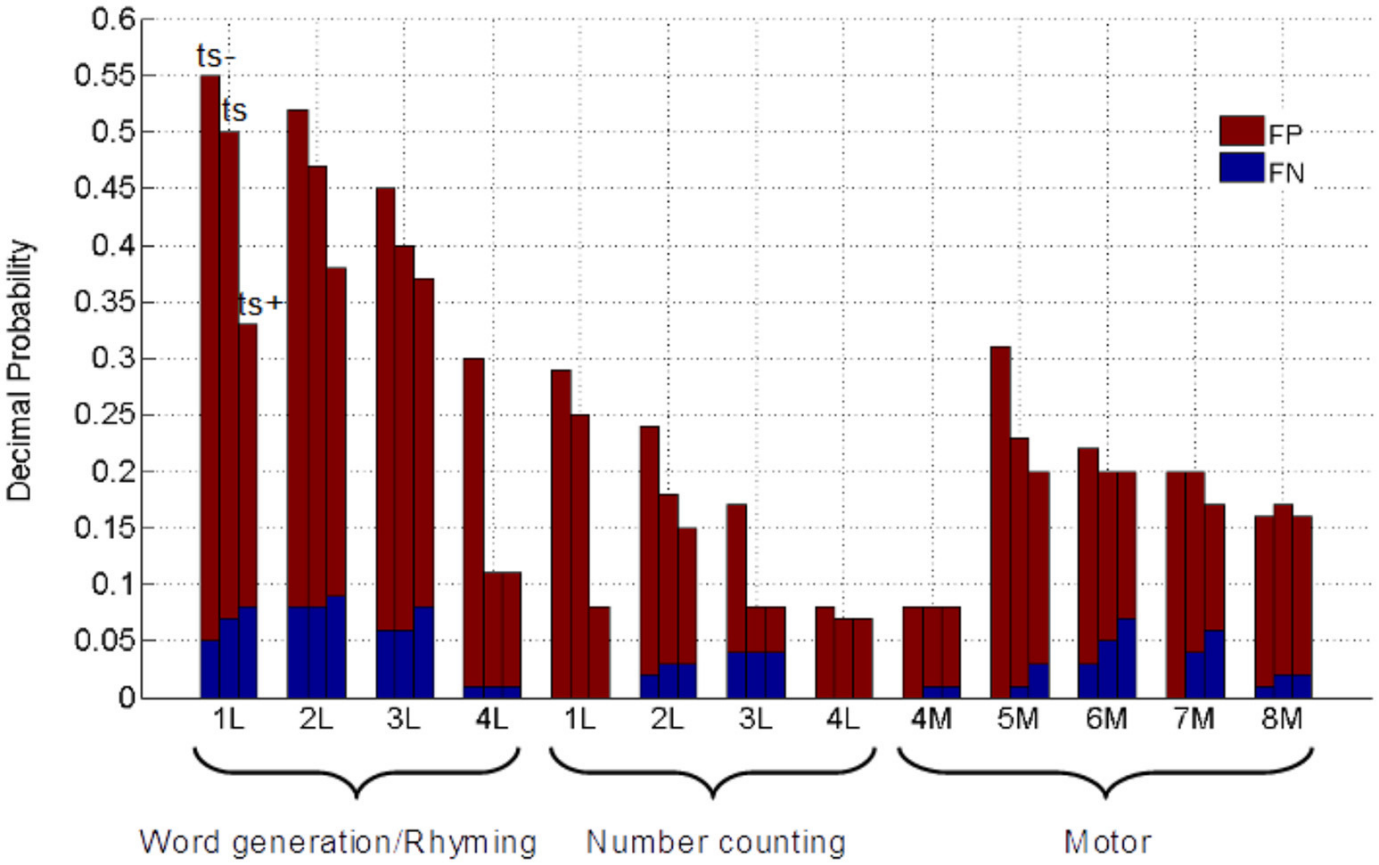
**Decimal probabilities of false positive (FP) and false negative (FN) error**. Patients are labeled according to Table 1, with motor and language tasks distinguished from language tasks by letters “M” and “L,” respectively. Grouped columns represent thresholding at t_s_−, t_s_, and t_s_+, as labeled.

The proportions of FP and FN that comprise the normalized total error are subsequently shown in Figure 5. It is evident that total error is dominated by FP occurrences, in a manner that is task-dependent as well as patient-dependent even at a fixed threshold (t_*s*_), thus explaining the horizontal spread of the data that drives the correlations observed in Figure 3. The FP occurrences for word generation/rhyming (0.28 ± 0.14), were significantly greater than for motor responses (0.15 ± 0.06; *p* = 0.01) and number counting (0.13 ± 0.08; *p* = 0.02). No significant differences in FP occurrences were found between motor responses and number counting. The FN values remained at a low level across all tasks (word generation/rhyming: 0.05 ± 0.03; motor: 0.02 ± 0.02; number counting: 0.02 ± 0.02). The FP values consistently decreased in patients across tasks when the fMRI threshold was increased, whereas a weaker trend toward increased FN values was also observed.

#### Task standardization and intraoperative task selection

**Figure 6:**
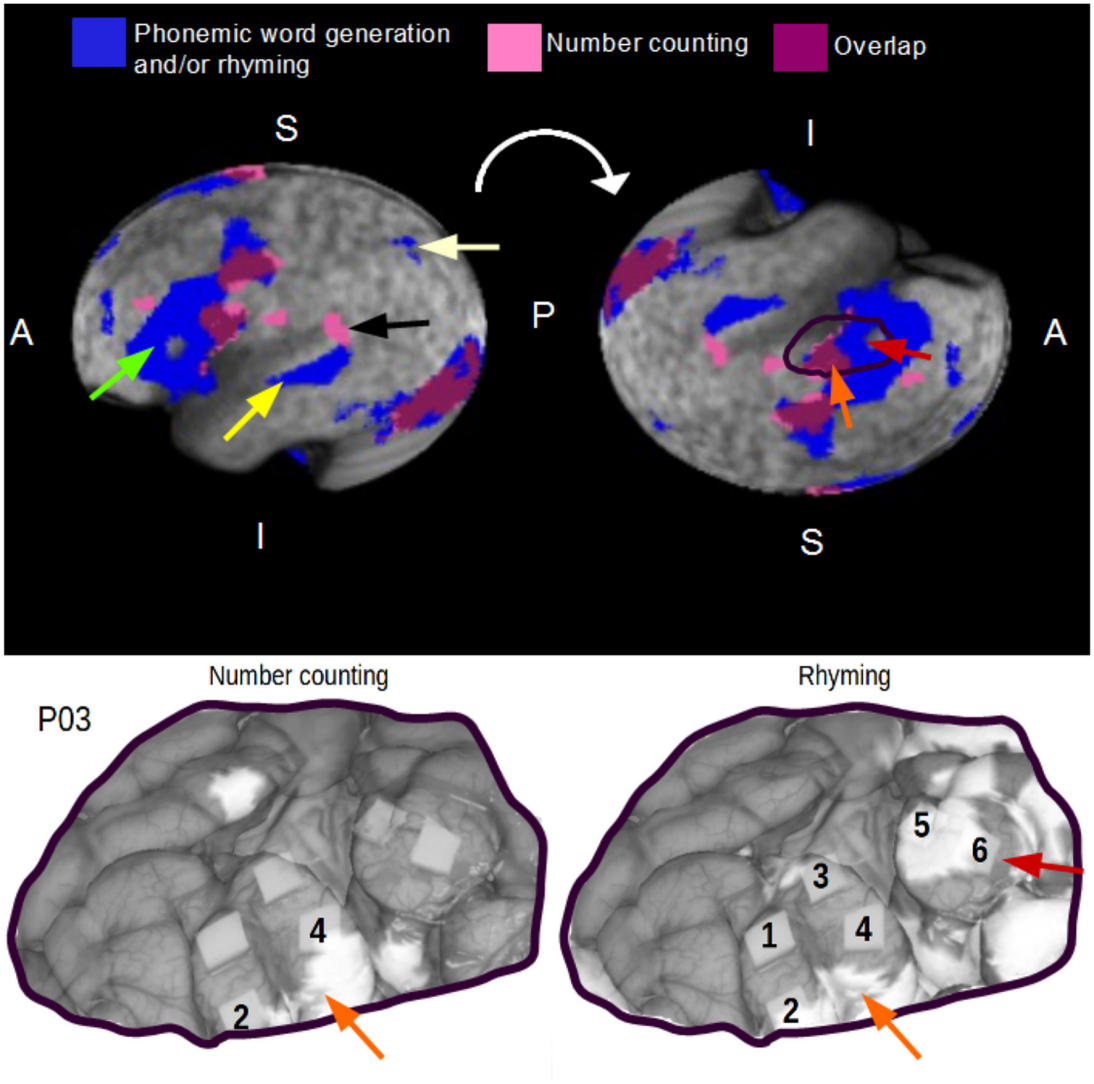
**(Above) Overlap of healthy control, group fMRI activity maps for the number counting task and a conjunction map of rhyming and phonemic word generation tasks**. Green, yellow, black, and pale yellow arrows correspond to unique anatomical locations of interest (further detail is provided in Section Task Standardization and Intraoperative Task Selection). A, P, S, and I correspond to anterior, posterior, superior, and inferior directions, respectively. **(Below)** Anatomical locations denoted by the orange and red arrows demonstrate parallel findings in the healthy control group and case data for patient P3.

Healthy control group fMRI data (Figure 6) revealed substantially different activity maps for the number counting task (pink) vs. a conjunction map of rhyming and phonemic word generation (blue). Overlap (purple) was observed in regions of the visual cortex and posterior frontal lobe, whereas large regions of the left frontal lobe (green arrow) and superior temporal gyrus (yellow arrow) were engaged by phonemic word generation/writing but not by the number counting task. Much smaller focal areas (e.g., black arrow) showed the reverse relationship. The superior parietal lobule (pale yellow arrow), associated with writing (Golestanirad et al., 2015), among other cognitive functions, was also activated by the word generation and rhyming tasks but not number counting. The Jaccard coefficient was found to be 0.11, suggesting that the overlap of task-related brain activity was low.

The group fMRI data were an important reference for interpreting the preoperative and intraoperative mapping results in patients. Figure 6 shows a representative example involving the co-registered fMRI and DCS maps for patient P3 performing number counting and rhyming, respectively. For DCS mapping with the number counting task, sites 2 and 4 in the posterior frontal gyrus were predicted by preoperative fMRI. For the rhyming task, sites 2 and 4 were again identified by DCS but only site 2 was predicted by fMRI. The region of fMRI activity lying superior to site 4 was also observed for the number counting task and corresponds to an similar area of overlap in the group fMRI data from healthy controls (orange arrows). In addition, anterior sites 5 and 6 evoked language errors with DCS during rhyming that were also predicted by preoperative fMRI with the analogous standardized task. These areas were confirmed from the fMRI group maps to be part of the common network of brain activity associated with rhyming and/or phonemic word generation in healthy controls (red arrows). Site 3 identified by DCS was also predicted by preoperative fMRI but was not observed in the group map. For completeness, it is also important to indicate that there were areas of disagreement between DCS and fMRI, notably two focal FP sites during number counting. For rhyming, sites 1 and 4 were FN areas of the patient fMRI map, whereas extensive FP areas on the superior and anterior margins of the craniotomy window were consistent with the fMRI group map results.

In general, intraoperative patient data showed fewer language errors evoked through number counting, in comparison to word generation/rhyming (Table 1). For example, in two patients (P1, P4), zero response(s) were generated during number counting despite findings of speech arrest during phonemic word generation. When standardized fMRI and DCS mapping of number counting were compared in representative patient case data (P3), sensitivity and specificity were 0.52 ± 0.03 and 0.92 ± 0.06, respectively. Standardized mapping of rhyming in the same patient yielded sensitivity and specificity measures of 0.69 ± 0.05 and 0.59 ± 0.07, respectively. Adoption of the non-standardized approach (i.e., fMRI of number counting was compared with intraoperative mapping of rhyming), often used in the literature, resulted in a significantly lower measure of sensitivity at 0.18 ± 0.01 and a higher specificity of 0.90 ± 0.07. For the other permutation (i.e., fMRI of rhyming was compared with intraoperative mapping of number counting) sensitivity was reduced to 0.61 ± 0.06, whereas specificity equaled 0.57 ± 0.06. Thus, sensitivity and specificity were affected by non-standardized task comparisons.

## Discussion and conclusions

Factors that cause variability in concordance between preoperative fMRI and intraoperative DCS maps of brain activity have been quantified in a cohort of glioma patients (WHO grades I-III). The results, as discussed in detail below, have important implications for how preoperative fMRI maps should be generated and used to inform intraoperative DCS, adding substantially to the pertinent scientific literature.

The concordance between fMRI and DCS results was found to vary with the behavioral task administered. Agreeing well with much of the previous literature (Tomczak et al., 2000; Bizzi et al., 2008; Giussani et al., 2010; Meier et al., 2013), the motor task produced sensitivity and specificity measures superior to the language tasks, and demonstrated less variation across patients. The differences arose primarily from a larger spread of error (FP + FN) and slightly lower, more variable TP values during language tasks than during the motor task. To understand how these effects might arise, the biophysical principles that underlie fMRI and DCS require some discussion.

Functional MRI is a neuroimaging technique that generates maps of brain activity by measuring Blood Oxygen Level Dependent (BOLD) signal changes during behavioral task performance. As electrophysiological activity is inferred indirectly and BOLD signals are weak, these issues can have a direct effect on activation map results. Tumor-induced neurovascular uncoupling has been reported in specific cases (Hou et al., 2006) and activation maps are influenced by the need to perform tasks consistently in repeated fashion to improve statistical power (Williams et al., 2014); by the choice of activation analysis method and statistical threshold (Monti, 2011; Churchill et al., 2012); and by sensitivity to various artifacts especially small head motions (Seto et al., 2001). A number of these factors produce FN activity, although some also produce FP activity (e.g., task-correlated head motion). However, fewer can explain the increase in FN activity observed in language tasks compared to motor tasks. A plausible candidate is related to ongoing, involuntary cognitive activations during the control block (primarily when rest is used as a task control) that can lead to a reduction in the number of voxels activated and thus increase FN occurrences (Hertz-Pannier et al., 2014; Stippich, 2015). Motor tasks involve continuous movements that are voluntarily controlled; ongoing movement during the control block can be easily identified. This is not the case for language tasks, however, especially phonemic word generation where responses were patient-controlled and rest was used for task contrast, thus subjecting the brain to “free thinking.” Although rhyming was contrasted with a task to control for activation related to sensory input and executing tablet responses (Table 2), there is the possibility of ongoing cognitive activity during the control task, such as self-evaluation of task performance involving a language component. It is also worth noting that task-uncorrelated motion (typical of patient populations and lengthy scan times) can also yield FN occurrences (Harrington et al., 2006).

Furthermore, fMRI can be considered a “passive” method in the sense that the mapping procedure has no influence on behavior. Multiple regions are typically shown to be active during task performance (in contrast with a baseline or control task according to the “cognitive subtraction” principle; Amaro and Barker, 2006) and *a priori* information is required to determine their respective functional roles. It is not necessarily known from this approach whether a specific region is essential or non-essential for task execution (Kekhia et al., 2011). For example, in broader semantic and phonological decision tasks such as rhyming, activity of non-essential brain regions has been shown including: the fronto-orbital cortex, superior frontal gyrus, cingulum, temporal fusiform gyrus, inferior temporal gyrus, superior parietal lobule, precuneus; as well as regions in the occipital lobe (Gutbrod et al., 2012). On the other hand, simple motor tasks tend to produce more focal activity (Grodd et al., 2001), though non-essential brain regions relating to visual or auditory processing of task instructions may be apparent in activation maps. In contrast, DCS can be considered an “active” mapping process that involves stimulation of a brain region while simultaneously evaluating neurological function. An inhibitory or excitatory behavioral response is elicited only if the stimulated region plays an essential role in task performance (Kekhia et al., 2011). When comparing the two mapping methods, therefore, it is not unexpected that fMRI will demonstrate considerable FP activation vs. DCS, and that for the specific language and motor tasks chosen, the former produces more FP activation with more variability. It is also likely that FP activation is less of a concern than FN activation, from a practical standpoint. As DCS remains the gold standard and preoperative fMRI is used to guide and assist surgical decisions, it is sufficient that fMRI maps identify candidate areas of brain activity lying nearby or adjacent to the tumor with high sensitivity and spatial accuracy. Any over-compensation of fMRI (e.g., due to activity from non-essential regions) can be assessed during the DCS procedure.

The present study also addresses the impact of task standardization and appropriate selection of intraoperative tasks on fMRI sensitivity and specificity outcomes. It was demonstrated in patients (with supporting healthy control group data) that a traditional intraoperative task such as number counting yields fMRI activity patterns distinctly different from phonemic word generation and rhyming tasks, and that number counting requires less regional engagement of critical language areas. This result was confirmed by DCS mapping in patients. It was also shown by example for patient 3, that lack of task standardization can have a major impact on fMRI sensitivity and specificity. Though not shown for brevity, this statement also holds for the other patients that underwent language mapping.

Others have also raised concerns about the lack of standardized tasks across the pre- and intra-operative testing environments (De Witte and Mariën, 2013), and the present data suggest that the sensitivity of fMRI is presently undervalued in the literature due to poor task standardization. Furthermore, the present data also support that use of traditional OR tasks (e.g., number counting) in the absence of more sophisticated paradigms limits the utility of DCS mapping, in addition to other technical and electrophysiological factors (e.g., patient cooperation, current intensity, effects due to after-discharge activity, and neuron refractory period; Rutten et al., 1999, 2002; Fernández Coello et al., 2013). Although number counting is typically used to assess speech articulation in a simple and time-efficient manner (Rofes and Miceli, 2014), limitations include the lack of ecological validity (Serletis and Bernstein, 2007), and lack of ability to localize critical language regions (Petrovich Brennan et al., 2007; Morrison et al., 2015). Neurosurgeons can improve language mapping through the use of more sophisticated paradigms within a standardized intraoperative task battery geared toward patient-specific characteristics (e.g., tumor location, preoperative deficits, and anatomo-functional correlations revealed by preoperative neuroimaging). Detailed protocols for developing robust individual task batteries have been previously reported in the literature (Fernández Coello et al., 2013).

Turning to other issues that can potentially affect the agreement of fMRI and DCS results, it is important to consider the procedure by which fMRI activity is spatially transformed to the surgical field. In the present work, a semi-automatic co-registration algorithm based on MI was used to align the 2-D projection of surface-rendered fMRI maps with the analogous 2-D optical projection of the brain surface. Co-registration errors were quantified using anatomical landmarks and were found to be well below the spatial resolution of both mapping methods, with negligible impact on concordance findings. Notably, however, brain shift was neither explicitly measured nor corrected, although the 2-D projection approach provides some inherent attenuation of spatial effects arising from expansion of the brain outward from the skull cavity. Previous studies have used both manual and semi-automated methods to co-register fMRI and DCS results in heterogeneous patient cohorts, demonstrating relatively high measures of sensitivity, and specificity without explicitly correcting for brain shift (FitzGerald et al., 1997; Lehéricy et al., 2000; Roux et al., 2003; Kuchcinski et al., 2015). Although brain shift correction methods improve co-registration further, especially in cases of large tumor volumes, the most pressing need in the present context involves development of real-time brain shift corrections to enhance intra-operative usage of preoperative fMRI data, to improve guidance of the DCS probe during stimulation (Berkels et al., 2014; Reinertsen et al., 2014).

Irrespective of such future developments, the conventional DCS mapping procedure involves photographing surgical chips that represent areas of functional significance on the brain surface. In the present work, the photographs were subsequently gridded at appropriate spatial resolution to mimic the true intraoperative mapping procedure and dimensions of the DCS probe (5 mm inter-electrode spacing), although the grid placement was arbitrary. This was subsequently determined to introduce negligible variations. Less than 1% deviations in fMRI sensitivity and specificity were identified as a result of arbitrary grid placement when sampling the co-registered surface data (Table 4). Kuchcinski et al. (2015) recently employed this gridding method for language mapping using a more stringent criterion of 1 × 1 × 1 mm voxels for spatial agreement between fMRI and DCS, and reported an fMRI sensitivity of 0.58 and specificity of 0.81 averaged across three language tasks (Kuchcinski et al., 2015). The reduced sensitivity and increased specificity in comparison to the present language results (i.e., Table 3: sensitivity of 0.66 and specificity of 0.74) is most likely a consequence of using a more strict matching criterion in the previous study, as it has been shown that when the criterion for fMRI agreement is loosened, sensitivity increases while specificity decreases (Pouratian et al., 2002). The use of less-stringent criteria in the present study is likely more appropriate to quantify agreement given that spatial resolution of DCS is not well defined; stimulation can induce an action potential in adjacent neurons extending millimeters beyond a focal area (Histed et al., 2009).

Considerable fMRI research has been undertaken previously to study the effect of varying and optimizing the statistical threshold for reporting fMRI maps from single subjects, as well as test-retest reliability studies (Bennett and Miller, 2013; Gorgolewski et al., 2013; Stevens et al., 2013; McKinsey et al., 2015; Morrison et al., 2016). However, the fraction of studies that directly explore such issues in relation to DCS maps in patients is small, and limited to reporting measures of sensitivity and specificity. The inclusion of TP, TN, FP, and FN plots in this study provides insight to individual patient trends, within- and between-patient variations, and the relative contributions of TP, TN, FP, and FN, that ultimately drive sensitivity and specificity outcomes. These data enable a more comprehensive assessment of factors influencing concordance, and several summarizing statements can be made after detailed consideration of Figures 3, 5. First, because DCS results are very focal, the proportions of TP and FN will be small (In particular, FN was 0.09 or less across all tasks and patients). Second, because the proportions of TP, TN, FP, and FN must total 1 and TP is small, an inverse relationship must exist between TN and total error (FP + FN), irrespective of the task. Third, FP values are the predominant source of spatial error between fMRI and DCS maps, occurring on a task-dependent and patient-dependent basis, as expected due to the underlying neural networks and biological variability.

When the statistical threshold of fMRI maps was increased toward more stringent *p*-values in the present study, FP measures (and thus sensitivity) decreased while TN measures (specificity) increased. This trend, as observed in Figures 3–5, agrees well with previous studies in the literature that have reported sensitivity and specificity across multiple thresholds (Rutten et al., 2002; Roux et al., 2003; Meier et al., 2013; Kuchcinski et al., 2015). Given this inverse relationship between sensitivity and specificity, both cannot be maximized simultaneously. In a preoperative setting without confirmatory evidence from DCS, determining the optimal threshold is challenging. To avoid misinforming the surgeon, active clusters nearby, or adjacent to the tumor site should be carefully scrutinized across multiple thresholds on an individual basis.

The technical factors addressed in this study, augmented by clinical/behavioral (e.g., age, handedness, Karnofsky performance status) and histopathologic factors (e.g., tumor grade and sub-type), collectively contribute the variability in concordance measured within- and across-studies. Although the latter categories were not explicitly investigated here, previous studies have reported moderate associations between tumor grade, tumor sub-type (specifically astrocytoma) and fMRI concordance (Bizzi et al., 2008; Kapsalakis et al., 2012; Kuchcinski et al., 2015), with WHO grade IV tumors exhibiting some of the lowest sensitivity measures (Bizzi et al., 2008). In addition, patients with good Karnofsky performance status (typically those with low-grade tumors) have been shown to yield higher measures of concordance (Kapsalakis et al., 2012). This is valuable information that can be used initially to predict the quality of patient fMRI data from a broad perspective. However, preoperative fMRI data is ultimately evaluated on an individual patient basis. Within-subject variations in the quality of fMRI activity maps have been strongly associated with subject motion in the scanner, as well as underlying cognitive processes associated with the chosen behavioral task, more so than technical details of data processing (Gorgolewski et al., 2013). This is evident in Figure 3, where within-patient data is more greatly distributed for different language tasks than for changes in fMRI threshold (as well as grid placement). Nonetheless, all sources of within-patient variability should be accounted for because they are likely to be additive. Preoperative fMRI data quality can only be optimized to a finite extent, therefore there is a need for a more rigorous screening protocol that assesses and scores data quality using knowledge provided from this study and prior literature. The current results, augmented by patient recruitment, will enable a large-scale study comprehensively investigating both the technical and biological sources of variability influencing concordance, and ranking the factors according to their relative effect.

## Author contributions

Study conception and design: MM, SG, SD, TS. Acquisition of data: MM, SD, FT, MG, GH, MC. Analysis and interpretation of data: MM. Drafting of manuscript: MM. Critical revision: MM, SG, SD.

## Disclosure

SG, FT, and TS have been issued a patent for the invention of the tablet technology. The primary authorMMand coauthors SG, TS and SD have just filed a provisional US patent application covering use of the tablet systemin neurosurgery.

### Conflict of interest statement

The authors declare that the research was conducted in the absence of any commercial or financial relationships that could be construed as a potential conflict of interest.
